# A tool for assessing sexual knowledge of people with Intellectual disabilities in Norway

**DOI:** 10.3389/fpsyt.2024.1330723

**Published:** 2024-03-19

**Authors:** Gøril Brevik Svae, Peter Zachariassen, Wenche Fjeld, Erik Søndenaa

**Affiliations:** ^1^ Department of Neurohabilitation, Oslo University Hospital, Oslo, Norway; ^2^ Division of Clinical Neuroscience, Department of Research and Innovation, Oslo University Hospital, Oslo, Norway; ^3^ Institute of Clinical Medicine, University of Oslo, Oslo, Norway; ^4^ Inland Norway University of Applied Sciences, Evenstad, Norway; ^5^ Department of Mental Health, Norwegian University of Science and Technology, Trondheim, Norway; ^6^ Centre for Research and Education in Security, Prisons and Forensic Psychiatry, Forensic Department Østmarka, St. Olav’s Hospital, Trondheim, Norway

**Keywords:** intellectual disability, sexual knowledge, assessment, psychometrics, scale

## Abstract

**Background:**

Previous research has shown that people with intellectual disabilities have limited sexual knowledge, which can be essential for developing positive sexuality. This study aimed to check the validity and reliability of an assessment tool (SexKunn) for measuring the sexual knowledge of people with intellectual disabilities in Norway. Further, we aimed to identify potential knowledge gaps among the participants and highlight their potential impact

**Methods:**

37 people with intellectual disabilities completed one assessment. 20 participants then completed a retest. Clinicians’ (n=23) views on the assessment tool were measured to obtain face validity. Positive and negative agreement analysis was used to identify potential dimensions in the data.

**Results:**

A weighted Cohen’s kappa for the test and retest of the overall scores was considered to be substantial. The clinicians described an acceptable face validity regarding high positive value scores and low negative burden scores. They also suggested improvements. The study identified that the participants lacked knowledge about female genitals, hygiene, appropriate touching/sexual acts with friends/intimate partners, and contraception.

**Conclusions:**

The SexKunn is a feasible assessment tool to measure sexual knowledge in people with intellectual disabilities. A lack of sexual knowledge of people with intellectual disabilities may violate their sexual rights.

## Introduction

People with intellectual disabilities are entitled to healthy sexuality, just like everyone else (1). In recent years, there has been a positive shift in attitudes regarding the sexual rights of people with intellectual disabilities (2). The internet has had a significant influence on many people’s sexual lives, and, unfortunately, has introduced a risk of sexual abuse for people with intellectual disabilities (3). However, one positive effect has been the growing visibility and acceptance of different sexual orientations and identities (LGBTQ), which people with intellectual disabilities may identify with (4). Increasing sexual openness and understanding can also include acknowledgement of the sexuality of people with intellectual disabilities. Several researchers have found that the sexual knowledge of people with intellectual disabilities is insufficient (5–7). For example, Isler, Tas (8) found that many individuals with intellectual disabilities knew little about body parts and lacked knowledge about male and female differences in the reproductive organs. Hole, Schnellert (9) also found that self-advocates with intellectual disabilities pointed out their own lack of sexual education. In cases where education was offered, it focused on risks, biology, and sexual consent, but not positive sexuality (9). Professional stakeholders believe that harmful sexual behaviour and abuse could be avoided if people with intellectual disabilities were given the opportunity to acquire sexual knowledge (10).

Despite the methodological limitations for research on sexual abuse among people with intellectual disabilities, such as small study sizes, we are certain that, as a group, they are exposed to an increased risk of sexual abuse compared to people without disabilities (11). A large register-based cohort study of people born in Sweden 1980–1991 (n = 1 232 564) concerning associations of intellectual disabilities with sexual offending and victimization, stratified by comorbid autism and attention deficit hyperactivity disorder (ADHD) have recently been published (12). The study results showed that the relative risks of sexual offending and victimization were elevated in men and women with intellectual disabilities without comorbidities (HRs 2.6–12.7). The highest risks for sexual offending in men (HRs 9.4–11.0) and for sexual assault victimization in women (HRs 11.0–17.1) related to intellectual disabilities and comorbid ADHD (12). A review by Tomsa, Gutu (13) found an overall prevalence of sexual abuse of 32.9% among people with intellectual disabilities. Further analysis showed that sexual abuse was higher among individuals living in institutions, and that a peer with an intellectual disability often committed the sexual offence. There are undoubtedly many complex explanations behind these findings. Still, there is widespread agreement that information about sex and sexual health should be more accessible for people with intellectual disabilities (14). Prevention programs can improve the knowledge of people with intellectual disabilities (15, 16), however some research has shown that preventive knowledge may not influence behaviour (15, 17).

In other words, we should be interested in what people with intellectual disabilities know - and do not know - about sexuality, both in a narrower (anatomical/physiological) and in a broader (relational/societal) sense. Kramers-Olen (18) points out a lack of published literature on psychometric assessment tools investigating sexual knowledge among people with intellectual disabilities. Paulauskaite, Rivas (19) also emphasise the urgent need for standardised validated assessment tools for this population. In recent years several new psychometric tests, which have been developed by a Spanish research group, have shown promising results. These include self-reported measure (the Detection of Sexual Abuse Risk Screening Scale; DSARss (20); the Inventory of Sexual Knowledge of people with Intellectual Disabilities; ISK-ID (21); the SExual BEhaviour and COncerns of people with Mild Intellectual Disabilities; SEBECOMID-S (22). The research group have also developed measures to assess the perceptions of other people (i.e. the parents perceptions of their child’s sexuality (23, 24) and as well as professional caregiver’s perceptions of their client’s sexuality (25). In this article, we propose that acquiring sociosexual knowledge can help protect individuals from exploitation and abuse, and therefore that the assessment of the sexual knowledge of people with intellectual disabilities is essential for adapting educational interventions.

### The aim of the study

The SexKunn assessment tool was developed to assess awareness and knowledge of the body, sexuality, emotions, and relationships (26). It was accessible to Norwegian professionals in its original form in 2002 (27). The tool’s purpose is to help clinicians better understand their clients/patients, allowing for adjustment and adaptation depending on their level of functioning. Most importantly, SexKunn should identify where knowledge is lacking, and thus where teaching and guidance about sexual health is required. In accordance with many other assessment tools, such as the Social-Sexual Knowledge & Attitudes Test (28), the Sexual Knowledge Interview Schedule (29), the Social Sexual Knowledge Assessment and Attitudes Tool – Revised (SSKAAT-R (30);, And the Sexual Knowledge, Experience and Needs Scale for People with Intellectual Disability (SEX KEN-ID (31);, SexKunn is supported with illustrations, it is intended to be helpful in assessing individuals with a lower level of verbal functioning (27). An updated version of SexKunn, based on comments from practitioners and people with intellectual disabilities, was completed in 2020 (32). SexKunn is the only clinical tool in use in hospital-based habilitation centres and community residences in Norway. We believe that the Sexkunn may be suitable for use in other countries as well. However, no research has been conducted on the updated SexKunn version, so it is necessary first to assess its measurement properties and clinical relevance.

We wanted to answer the following research questions:

RQ1: To what extent is SexKunn reliable and valid for measuring sexual knowledge among adults with intellectual disabilities? And what is its test-retest agreement?

RQ2: What is the face validity, feasibility, and utility of the SexKunn assessment tool?

RQ3: What knowledge gaps can be identified, and what impact can these gaps have?

## Methods

### The assessment tools

SexKunn was inspired by SEX KEN-ID by McCabe, Cummins (31). However, the questions and illustrations are unique and originally developed by assistant professor WF and psychologist PZ (co-authors), together with illustrator Anna Fiske. The first SexKunn version from 2002 underwent a lengthy revision process with feedback from people with intellectual disabilities, professionals using the assessment for clinical purposes, and developers. Throughout the process, the items and illustrations have been evaluated, and thus the Sexkunn has taken its current form. Feedback had suggested that there was a need for more detailed questions and pictures relating to the detection of and protection against harmful sexual behavior. Furthermore, it was felt that SexKunn needed to be visually less “gender dichotomous”, and finally, it should include some questions concerning the Internet and social media.

In its current form the SexKunn assessment tool includes a scale of 62 questions, associated black and white drawings, and instructions to point at the most relevant drawing or details. The drawings are simplified, leaving out irrelevant details, and are intended to appear as non-provocative and non-offending. These 62 questions are divided under seven subscales (1. Identity and body, 2. Puberty, 3. Hygiene, 4. Emotions and social relations, 5. Sexual behaviour, 6. Boundaries and abuse, and 7. Contraception and sexual education). Illustrations support 54 items; for example, the participants can directly point at a picture as an answer. Ten items are direct questions without illustrations.

To assess the face validity, feasibility and utility of the SexKunn assessment tool, an adjusted version of the QQ-10 questionnaire (33) was distributed online to an independent group of clinicians. The QQ-10 is a validated self-reported tool designed to measure the responder’s views on questionnaires (33). In the present study, each item reflected the clinicians’ perspectives on SexKunn, rated on a Likert scale from strongly disagree to strongly agree (coded as 0–4). The scores are summarized separately for the first six questions, comprising the *Value score*, and the next four questions comprising the *Burden score*. QQ-10 has possible scores ranging between 0 and 100 (0 being the worst and 100 being the best possible view of the questionnaire).

### Study design, participants, and procedure

#### The patient population

A cross-sectional design was used in a consecutive sample (n=37), and a proportion of the participants (n=20) were asked to retest to measure the test-retest reliability. The specialised health care department for people with intellectual disabilities (SHCS) at Oslo University Hospital in Norway initiated the evaluation of SexKunn. Nine clinical specialists working at an SHCS and having extended knowledge of intellectual disability conducted the interviews with the 37 participants. The clinical specialists were 6 women and three men aged between 35-60, having formal competence in the purpose and administration of the SexKunn assessment tool. Participants were offered the opportunity to be accompanied by a trusted person, such as a family member or staff member, for support during the interview, creating a safe atmosphere.

The SHCS departments in the Trøndelag, Innlandet and Østfold counties of Norway contributed to collecting test data. All participants were recruited from one of the regional SHCSs. All interviews were conducted in person, although they occurred during a period with pandemic restrictions. We strived to conduct a retest within 2-4 weeks. However, this was difficult in many cases because of sudden changes within covid-restrictions. Retests were conducted before participating (individually or in a group) in a sexual health education program. At all retests, the participants were asked if there had been any changes in their lives when it came to their sexual health to detect formal or informal sex education or exposure to sexual abuse The interviews lasted approximately 20 minutes, depending on breaks. See **Table 1** for a description of the participants.

**Table 1:** Gender, age, and level of functioning in the sample.

	Test (n=37)	Retest (n=20)
Gender Mean age (Sd) Age range Level of functioning Living with family Group home Living independently	19 M, 17 F, 1 other 28,3 (8,9) 18-58 33 Mild, 4 Moderate 11 23 2	8 M, 11 F, 1 other 28,6 (10,3) 18-58 17 Mild, 3 Moderate 6 12 2

#### The clinician’s population

A survey consisting of the QQ-10 and four additional questions concerning experiences using the SexKunn assessment tool was distributed online to a group of clinicians (n=23). The four questions were: 1) How many SexKunn assessments have you administered? 2) Do you have any suggestions for improving the assessment tool? 3) Are some important topics not covered in the assessment tool? and 4) Is there too much focus on certain topics in the SexKunn assessment tool?

### Ethics and data management

All participants or legal guardians gave written consent following adapted information about the study. The Regional Committees for Medical and Health Research Ethics of Norway approved the study (approval number 2018/2296). Economic compensation (600 NOK; approx. 50 Euro) for their time and any inconvenience was arranged for the participants recruited for the retest. SPSS and STATA were used for statistical analyses. There were many nominal items, so a test-retest agreement of the items was used to supplement the more frequently used calculated Cohen’s Kappa.

## Results

All the participants in the sample (n=37) had one assessment, and 20 participants were later assessed with a retest. As seen in **Table 1**, most participants had a mild intellectual disability.

The SexKunn assessment including all 62 items showed a high internal consistency (Cronbach’s α = 0.923). Across the seven subscales, the α was estimated at 0.827, which is considered a good internal consistency. However, the seven subscales disclosed limited correlations with each other (from 0.27 to 0.61), indicating that they measured different constructs.

A weighted Cohen’s kappa was estimated for the test and retest of the overall SexKunn scores, giving a kappa of 0.73, which is considered substantial. Measuring the positive and negative agreement conveys the relevant information and is helpful for informing clinical practice (27). One interpretation of the positive agreement is that, if a test rates a variable with “yes”, it is probable that a retest based on the same test subject will come to the same conclusion. A negative agreement can be interpreted in the same way. A further analysis of the agreements of all 62 SexKunn items resulted in disagreements from test to retest for certain items (see **Appendix**).

The clinicians’ views on the measure, assessed with the QQ-10 questionnaire, showed acceptable ratings regarding the Positive Value-score and the Negative Burden-score of the SexKunn assessment tool. The overall mean Positive Value-score was high (range 52–100; mean 89), and the mean Negative Burden-score was low (range 4–39; mean 18). **Table 2** shows the clinicians’ views on the SexKunn assessment tool evaluated with QQ-10, including the percentage and count for each response (*n* = 23 clinicians).

**Table 2:** Clinicians’ views on the SexKunn assessment tool assessed with QQ-10, including percentage and count for each response (*n* = 23 clinicians).

Statement		Strongly disagree	Mostly disagree	Neither agree nor disagree	Mostly agree	Strongly agree
*Value*						
*Improved communication*		0	4.3 (1)	4.3 (1)	60.9 (14)	34.8 (8)
*Relevant*		0	0	0	47.8 (11)	52.2 (12)
*Ease of use*		0	0	0	52.2 (12)	47.8 (11)
*Comprehensive*		0	8.7 (2)	39.1 (9)	47.8 (11)	4.3 (1)
*Enjoyable*		0	0	8.7 (2)	47.8 (11)	43.5 (10)
*Happy to complete again*		0	0	0	30.4 (7)	69.6 (16)
*Burden*						
*Too long*		8.7 (2)	52.2 (12)	39.1 (9)	0	0
*Too embarrassing*		56.5 (13)	39.1 (9)	4.3 (1)	0	0
*Too complicated*		56.5 (13)	26.1 (6)	8.7 (2)	4.3 (1)	4.3 (1)
*Upsetting*		73.9 (17)	13.0 (3)	8.7 (2)	4.3 (1)	0

### Internal consistency

A majority of the clinicians had administered a large number of SexKunn assessments, and 59.1% (n=13) had used it more than 10 times. Some suggestions for improvements to the tool were reported. Most of these comments concerned the layout, confusing scoring instructions, ambiguous terms, and suggestions for digitalising the tool. Several clinicians pointed out that SexKunn does not address different aspects of issues related to the internet and that it needs better questions about sexual consent, a crucial topic for assessing sexual knowledge in people with intellectual disabilities. Some clinicians described an overfocus on gender, age, and body parts.

### Limited knowledge and test-retest disagreement

Half or more of the participants scored wrong according to the scoring manual on the test and retest on several items (See **Appendix**). Some missing knowledge was found in most subscales, and especially for the subscales “Hygiene” (3 items), “Sexual behavior” (3 items), and “Contraception and sexual education” (4 items) in which most of the erroneous scores were observed (See **Table 3**). A changed response from test to retest was found in several items, with no dominant subscale.

**Table 3:** Subscales and items with erroneous scores at the SexKunn assessment tool (both test and retest).

Subscale	Item	Wrong answer
Identity and body	**Q5b Naming the vaginal opening and the clitoris**	10 (50%)
Puberty	**Q11 When is adulthood**	13 (65%)
	**Q15 Body changes with age**	12 (60%)
Hygiene	**Q18 Reason for washing**	14 (70%)
	**Q19 Reason for wearing clean clothes**	12 (60%)
	**Q22 Reason for brushing your teeth**	13 (65%)
Sexual Behaviour	**Q34 What do friends do together**	15 (75%)
	**Q37 How to touch a lover**	10 (50%)
	**Q38 Who is having sex**	10 (50%)
Boundaries and abuse	**Q43: Who can she have sex with**	10 (50%)
	**Q44: Who can he have sex with**	11 (55%)
Contraception and sexual education	**Q55 How can a girl avoid pregnancy**	11 (55%)
	**Q56 How does a girl know that she is pregnant?**	16 (80%)
	**Q57 What can a girl do if she doesn’t want to have the baby?**	11 (55%)
	**Q62 Where can you learn more about sex?**	12 (60%)

## Discussion

This study aimed to check the reliability, validity, and the test-retest agreement of SexKunn, an assessment tool for examining the sexual knowledge of people with intellectual disabilities.A further goal was to identify measured knowledge gaps and look at their potential impact. The results showed an overall substantial test-retest agreement and that the SexKunn assessment tool is of clinical value in Norway. Moreover, as measured by SexKunn, the participants struggled to answer questions about female genitals, people’s ages, appropriate touching/sexual acts with friends/intimate partners, and contraception.

### To what extent is SexKunn reliable and valid and what is its test-retest agreement?

SexKunn is a 62-item scale designed to help clinicians assess the sexual knowledge of clients with intellectual disabilities. There was substantial test-retest agreement overall. The test results show that certain items have a high level of agreement among all participants, and some items have a very low level of matching, meaning they are too random. The weighted kappa for the test and retest of the overall scores was considered substantial. However, measuring the agreement revealed significant changes in certain items. According to Lydersen (34), measuring agreement is preferable to using Cohen’s kappa because, at a low prevalence [i.e., correct answers in most assessments of both measurements (pre and post)] and a high degree of agreement, Cohen’s kappa will always be paradoxically low. This does not occur to the same extent for specific agreement, where the negative correlation will typically be high, while positive correlation may be moderate. Measuring positive and negative agreement has great clinical relevance, although these methods have not been widely adopted (34, 35). Assessment of certain items, such as 12, 13, 21, 22, and 28, resulted in a low positive and negative agreement. For example, for item 13, six participants’ answers were not in accordance with the scoring manual on the test and retest, and five participants’ answers changed from incorrect on the test to correct on the retest. These variations result in unstable measurements, and there may be several explanations for why they occur. It may be that an item fails to capture a participant’s knowledge, or that they simply do not know the answer. The participant’s knowledge may also have changed from test to retest. Finally, there are differences in the number of potential points for many items – 1 point for one correct answer and 2 points for elaborating further. These point variations may make the results seem random when compared.

Of the 62 items, 17 were scored more than 95% correctly in accordance with the scoring manual in both the test and retest. This was the case for eight out of eleven items in subscale 4: Emotions and social relations (see **Appendix**), resulting in minor response variations. It seems that these items have little effect on variations in the measured sexual knowledge. One reason for this may be that our selected participants were very high functioning, and all answered correctly according to the scoring manual. Those participants who scored poorly on the first test also scored poorly on the retest.

This study showed a very high Cronbach’s α for all the included SexKunn items. Cronbach’s α is a common measure of reliability (36), and, similar to studies by Talbot and Langdon (37) and Galea, Butler (38), we calculated it for all included items. Using Cronbach’s α as the sole index of reliability is no longer sufficient, and other indices of internal consistency must be reported as well (39).

### The face validity, feasibility, and utility of the SexKunn assessment tool

The clinicians find SexKunn to be a appreciated first step in guidance with a limited burden, confirming that it is of clinical value in Norway. According to the clinicians, SexKunn lacks items about some aspects of online behaviour and sexual consent. Gil-Llario, Díaz-Rodríguez (40) found that people with intellectual disabilities expressed their sexuality online during the Covid-19 lockdown. Moreover, a study by Berget (41) found that young people with intellectual disabilities have more technical knowledge than their stakeholders, who lack the skills to give sufficient digital help. Online communication is changing rapidly, and it can be demanding for an assessment tool to keep up with developments. Still, assessments like SexKunn can facilitate the discussion of relevant situations where knowledge of sexual consent is important.

### What knowledge gaps can be identified, and what impact can these gaps have?

The findings revealed that the participants struggled to answer correctly according to the scoring manual on the topics of female genitals, hygiene, touching which is allowed and not allowed within certain relationships, potential sexual partners, and appropriate age differences. In addition, the participants struggled to answer items questioning contraception, pregnancy, and where to learn more about sexual education. An explanation for these results may be that the questions or illustrations are insufficient, or that the categories are easily misunderstood. However, a potential lack of knowledge about female genitalia may be connected with the overrepresentation of problematic sexual behaviour among males with intellectual disabilities, as illustrated by Tarnai (42). A lack of knowledge about a woman’s genitals can imply that people with intellectual disabilities may not care about their own or others’ sexual pleasure and expression, as outlined by Alexander and Taylor Gomez (43). Basic knowledge of the body and hygiene is closely linked to the ability to look after oneself, a social valuable skill. Having sexual knowledge is fundamental in empowering people with intellectual disabilities to navigate the sexual landscape and is closely linked to preventing sexual abuse (44). A lack of fundamental sexual knowledge may therefore limit the possibilities for people with intellectual disabilities’ to make informed decisions. Our results align with a study by Singh Shrestha, Ishak (44), showing that people with intellectual disabilities lack knowledge about sexual health and contraception, violating their sexual and reproductive rights.

Future investigations and directions should explore what teaching and guidance methods for people with intellectual disabilities that increase sexual knowledge and sexuality-related skills, in combination, can give efficient sexual education and can decrease the risk of being exposed to sexual abuse- or committing sexual abuse. Further work is needed to develop the psychometric properties of SexKunn. In that manner, we believe that it will be advantageous to investigate the work of Gil‐Llario, Castro‐Calvo (21), Gil-Llario, Fernández-García (24) further to succeed in this.

### Limitations

There are several limitations in this study. The first is the small sample size which limited the statistical findings, and thereby the study results. The recruitment process was challenging, with obstacles such as pandemic restrictions and the process of collecting consent agreements from the legal guardians of potential participants with intellectual disabilities. Moreover, the data material was collected through a convenience sample of patients at several SHCS and we did not have access to information on participant’s diagnoses, besides that of intellectual disability. The financial compensation of NOK 600 was directed to the bank account of the participants conducting the retest, and we can assume that money affected the motivation to participate in the study. However, it is hard to imagine that they could have influenced the quality of the responses that the participants gave in this study. Due to the low number of participants in a convenience sample, the results cannot be generalised. When interpreting our results, we must also consider biases, such as shame, as sexuality can be a sensitive topic. Another limitation may be that no background information about the clinicians is provided. However, we did not request this information since the study was only concerned with the feasibility of the SexKunn assessment tool. A potential bias is that the respondents were already acquainted with the authors who distributed the online link with the QQ-10 questionnaire, which could influence the clinicians’ answers. Still, the clinicians’ responses were not entirely positive, and many had suggestions for improvements.

## Data availability statement

The original contributions presented in the study are included in the article/**Supplementary Material**. Further inquiries can be directed to the corresponding author.

## Ethics statement

The studies involving humans were approved by The Regional Committees for Medical and Health Research Ethics of Norway. The studies were conducted in accordance with the local legislation and institutional requirements. Written informed consent for participation in this study was provided by the participants’ legal guardians/next of kin.

## Author contributions

GS: Writing – original draft. PZ: Writing – review & editing. WF: Writing – review & editing. ES: Supervision, Writing – original draft.
