# Supplementary material for: A tool for assessing sexual knowledge of people with Intellectual disabilities in Norway
Source: Front Psychiatry. 2024 Mar 19;15:1330723. doi: 10.3389/fpsyt.2024.1330723 (PMC10985616; doi:10.3389/fpsyt.2024.1330723)
Supplement: Supplementary file 1 [file DataSheet_1.pdf]

Appendix

Study of test-retest agreement in accordance with the scoring manual of the 62 items of the SexKunn assessment tool.

Theme 1: Identity and body

			Negative agreement	Positive agreement
**Q1: Who is a man**	Wrong (retest)	Right (retest)		
Wrong (test)	1	2		
Right (test)	0	17	0.5	0.94
**Q2: Who is a woman**	Wrong (retest)	Right (retest)		
Wrong (test)	2	2		
Right (test)	0	16	0.67	0.94
**Q3: Point to 14 bodyparts**	Wrong (retest)	Right (retest)		
Wrong (test)	6	1		
Right (test)	2	11	0.8	0.88
**Q4: Function of 9 bodyparts**	Wrong (retest)	Right (retest)		
Wrong (test)	6	5		
Right (test)	3	6	0.6	0.6
**Q5a: Naming female genitals**	Wrong (retest)	Right (retest)		
Wrong (test)	2	2		
Right (test)	2	14	0.5	0.88
**Q5b: Naming the vaginal opening and the clitoris**	Wrong (retest)	Right (retest)		
Wrong (test)	10	1		
Right (test)	3	6	0.83	0.75
**Q6a: Naming male genitals**	Wrong (retest)	Right (retest)		
Wrong (test)	1	0		
Right (test)	0	19	1	1
**Q6b: Naming the head of penis and the scrotum**	Wrong (retest)	Right (retest)		
Wrong (test)	5	1		
Right (test)	2	12	0.77	0.89

Theme 2: Puberty:

			Negative agreement	Positive agreement
**Q7: Identifying generations**	Wrong (retest)	Right (retest)		
Wrong (test)	0	1		
Right (test)	1	18	0	0.95
**Q8: Pointing to a child**	Wrong (retest)	Right (retest)		
Wrong (test)	0	2		
Right (test)	2	16	0	0.90
**Q9: Pointing to an adult**	Wrong (retest)	Right (retest)		
Wrong (test)	3	2		
Right (test)	0	15	0.75	0.95
**Q10: When is adolescence**	Wrong (retest)	Right (retest)		
Wrong (test)	1	3		
Right (test)	1	15	0.40	0.91
**Q11: When is adulthood**	Wrong (retest)	Right (retest)		
Wrong (test)	13	3		
Right (test)	3	1	0.81	0.25
**Q12: From boy to man**	Wrong (retest)	Right (retest)		
Wrong (test)	6	2		
Right (test)	1	11	0.80	0.88
**Q13: From girl to woman**	Wrong (retest)	Right (retest)		
Wrong (test)	6	5		
Right (test)	2	7	0.63	0.67
**Q14: Who is old**	Wrong (retest)	Right (retest)		
Wrong (test)	0	0		
Right (test)	0	20		1
**Q15: Body changes with age**	Wrong (retest)	Right (retest)		
Wrong (test)	12	3		
Right (test)	3	2	0.8	0.4
**Q16: Who can have a period**	Wrong (retest)	Right (retest)		
Wrong (test)	3	6		

Right (test)	2	9	0.43	0.69

Theme 3: Hygiene:

			Negative agreement	Positive agreement
**Q17: What parts of your body need to be washed every day**	Wrong (retest)	Right (retest)		
Wrong (test)	3	2		
Right (test)	1	14	0.67	0.9
**Q18: Reason for washing**	Wrong (retest)	Right (retest)		
Wrong (test)	14	1		
Right (test)	0	5	0.97	0.91
**Q19: Reason for wearing clean clothes**	Wrong (retest)	Right (retest)		
Wrong (test)	12	5		
Right (test)	1	2	0.80	0.40
**Q20: When to wash your hands**	Wrong (retest)	Right (retest)		
Wrong (test)	7	2		
Right (test)	1	10	0.82	0.87
**Q21: When to brush your teeth**	Wrong (retest)	Right (retest)		
Wrong (test)	2	3		
Right (test)	5	10	0.30	0.71
**Q22: Reason for brushing your teeth**	Wrong (retest)	Right (retest)		
Wrong (test)	13	3		
Right (test)	3	1	0.81	0.25

Theme 4: Emotions and social relationships:

			Negative agreement	Positive agreement
**Q23: Who is happy**	Wrong (retest)	Right (retest)		
Wrong (test)	0	0		
Right (test)	1	19	0	0.97

**Q24: Who is angry**	Wrong (retest)	Right (retest)		
Wrong (test)	0	0		
Right (test)	1	19	0	0.97
**Q25: Who is sad**	Wrong (retest)	Right (retest)		
Wrong (test)	0	0		
Right (test)	0	20		1
**Q26: Who is happy**	Wrong (retest)	Right (retest)		
Wrong (test)	1	0		
Right (test)	0	19	1	1
**Q27: How to identify happiness**	Wrong (retest)	Right (retest)		
Wrong (test)	0	0		
Right (test)	1	19	0	0.97
**Q28: How to support people who are sad**	Wrong (retest)	Right (retest)		
Wrong (test)	7	5		
Right (test)	3	5	0.58	0.50
**Q29: Who is jealous**	Wrong (retest)	Right (retest)		
Wrong (test)	0	0		
Right (test)	0	20		1
**Q30: Who are lovers**	Wrong (retest)	Right (retest)		
Wrong (test)	1	0		
Right (test)	0	19	1	1
**Q31: Who are friends**	Wrong (retest)	Right (retest)		
Wrong (test)	0	0		
Right (test)	1	19	0	0.97
**Q32: Who can be her lover**	Wrong (retest)	Right (retest)		
Wrong (test)	9	0		
Right (test)	3	8	0.86	0.84
**Q33: Who can be his lover**	Wrong (retest)	Right (retest)		
Wrong (test)	8	3		
Right (test)	3	6	0.73	0.67

Theme 5: Sexual behaviour:

			Negative agreem.	Positive agreem.
**Q34: What do friends do together**	Wrong (retest)	Right (retest)		
Wrong (test)	15	2		
Right (test)	2	1	0.88	0.33
**Q35: What do lovers do together**	Wrong (retest)	Right (retest)		
Wrong (test)	6	0		
Right (test)	5	9	0.71	0.78
**Q36: How to touch a friend**	Wrong (retest)	Right (retest)		
Wrong (test)	8	1		
Right (test)	4	7	0.76	0.74
**Q37: How to touch a lover**	Wrong (retest)	Right (retest)		
Wrong (test)	10	2		
Right (test)	2	6	0.83	0.75
**Q38: Who is having sex**	Wrong (retest)	Right (retest)		
Wrong (test)	10	1		
Right (test)	4	5	0.8	0.67
**Q39: Who is aroused**	Wrong (retest)	Right (retest)		
Wrong (test)	6	4		
Right (test)	2	8	0.67	0.73
**Q40: Who is masturbating**	Wrong (retest)	Right (retest)		
Wrong (test)	6	1		
Right (test)	2	11	0.8	0.88
**Q41: Who is having sexual intercourse?**	Wrong (retest)	Right (retest)		
Wrong (test)	4	0		
Right (test)	1	15	0.89	0.97
**Q42: Are any of these people having intercourse?**	Wrong (retest)	Right (retest)		
Wrong (test)	3	1		
Right (test)	1	15	0.75	0.94

Theme 6: Boundaries and abuse

			Negative agreement	Positive agreement
**Q43: Who can she have sex with**	Wrong (retest)	Right (retest)		
Wrong (test)	10	1		
Right (test)	1	8	0.91	0.89
**Q44: Who can he have sex with**	Wrong (retest)	Right (retest)		
Wrong (test)	11	1		
Right (test)	2	6	0.88	0.8
**Q45: Non-consensual sex**	Wrong (retest)	Right (retest)		
Wrong (test)	0	2		
Right (test)	0	18	0	0.95
**Q46: Who is having non-consensual sex**	Wrong (retest)	Right (retest)		
Wrong (test)	1	0		
Right (test)	0	19	1	1
**Q47: How can you tell**	Wrong (retest)	Right (retest)		
Wrong (test)	0	1		
Right (test)	2	17	0	0.92
**Q48: Where can you have sex**	Wrong (retest)	Right (retest)		
Wrong (test)	0	1		
Right (test)	0	19	0	0.97
**Q49: What is legal sex**	Wrong (retest)	Right (retest)		
Wrong (test)	7	4		
Right (test)	0	9	0.78	0.82
**Q50: What is illegal sex**	Wrong (retest)	Right (retest)		
Wrong (test)	5	4		
Right (test)	2	9	0.63	0.75
**Q51: Can adults have sex with children in movies or online**	Wrong (retest)	Right (retest)		
Wrong (test)	0	2		
Right (test)	0	18	0	0.95

**Q52: Can an adult and a child be in a online relationship**	Wrong (retest)	Right (retest)		
Wrong (test)	1	0		
Right (test)	0	19	1	1
**Q53: Who can have sex within a family**	Wrong (retest)	Right (retest)		
Wrong (test)	1	2		
Right (test)	1	16	0.4	0.91

Theme 7: Contraception and sexual education

			Negative agreement	Positive agreement
**Q54: How can a girl get pregnant**	Wrong (retest)	Right (retest)		
Wrong (test)	0	0		
Right (test)	0	20		1
**Q55: How can a girl avoid pregnancy**	Wrong (retest)	Right (retest)		
Wrong (test)	11	4		
Right (test)	1	4	0.81	0.62
**Q56: How does a girl know that she is pregnant?**	Wrong (retest)	Right (retest)		
Wrong (test)	16	3		
Right (test)	0	1	0.91	0.40
**Q57: What can a girl do if she doesn't want to have the baby?**	Wrong (retest)	Right (retest)		
Wrong (test)	11	3		
Right (test)	2	4	0.81	0.62
**Q58: What can a boy use to prevent getting a girl pregnant**	Wrong (retest)	Right (retest)		
Wrong (test)	1	0		
Right (test)	0	19	1	1
**Q59: Who is having involuntary sex**	Wrong (retest)	Right (retest)		
Wrong (test)	1	0		
Right (test)	0	19		

**Q60: How to use a condom**	Wrong (retest)	Right (retest)		
Wrong (test)	5	1		
Right (test)	3	11	0.71	0.85
**Q61: How to get a condom**	Wrong (retest)	Right (retest)		
Wrong (test)	10	0		
Right (test)	1	9	0.95	0.95
**Q62: Where can you learn more about sex?**	Wrong (retest)	Right (retest)		
Wrong (test)	12	2		
Right (test)	0	6	0.81	0.44
